# Field experiment and numerical simulation of point source irrigation with multiple tracers

**DOI:** 10.1371/journal.pone.0190500

**Published:** 2018-01-02

**Authors:** Tarek Selim, Fethi Bouksila, Yasser Hamed, Ronny Berndtsson, Akissa Bahri, Magnus Persson

**Affiliations:** 1 Civil Engineering Department, Faculty of Engineering, Port Said University, Port Said, Egypt; 2 National Institute for Research in Rural Engineering, Water, and Forests (INRGREF), Tunis, Tunisia; 3 Department of Water Resources Engineering, Lund University, Lund, Sweden; 4 Center for Middle Eastern Studies, Lund University, Lund, Sweden; 5 National Agronomic Institute of Tunisia, Tunis, Tunisia; The Education University of Hong Kong, HONG KONG

## Abstract

Dyes like Brilliant Blue have similar adsorptive behaviour as some organic contaminants, e.g., pesticides. Bromide ions, on the other hand, move much like NO_3_-N (fertilizer) in soil. Consequently, by using these two tracers, it is possible to in a general way mimic how organic contaminants and fertilizers may move through soils. Three plots with sandy soil in semiarid Tunisia were irrigated during three successive hours using a single irrigation dripper and high-saline solution (10.50 dS m^-1^) containing dye and bromide. Fifteen hours after cease of infiltration, horizontal 5 cm trenches were dug in the soil and dye pattern, bromide concentration, and soil water content were recorded. Preferential flow occurred to some degree, however, it did not dominate the solute transport process. Therefore, drip irrigation can be recommended to improve plant culture for a better water and soil nutrient adsorption. Numerical simulation using HYDRUS-2D/3D was performed to replicate the field experiments. Observed soil water contents before and after infiltration were used to run an inverse parameter estimation procedure to identify soil hydraulic parameters. It was found that for both field experiments and numerical simulations the mobility of bromide is different from the mobility of dye. The dye was retarded approximately twice by volume as compared to bromide. The simulation results support the use of HYDRUS-2D/3D as a rapid and labor saving tool for investigating tracers’ mobility in sandy soil under point source irrigation.

## Introduction

Improved irrigation efficiency can decrease irrigation amount in water scarce countries, especially in the Middle East. Drip irrigation is in general a way to improve irrigation efficiency and reduce harmful effects of irrigated agriculture on the environment. It offers a high degree of control leading to adequate water and fertilizer application according to crop requirements, thereby reducing leaching and pollution risk of shallow groundwater. In addition, it minimizes salinity and matrix stress in the root zone, though salts may accumulate in the periphery of the wetted area [1]. Higher levels of salinity in the irrigation water can be tolerated with drip irrigation as compared to other irrigation methods [2]. The magnitude of the soil water content is decreasing away from the point source. This results in a root distribution pattern in which most of the roots are typically found in the highly leached zone beneath the drippers [3]. Accurate estimation of wet bulb shape and dimensions is important for determining the number of emitters per plant and their locations in relation to the plant. The wet bulb dimensions depend mainly on soil structure and texture, drip flow, application frequency, and soil initial moisture [4]. It is generally accepted that water may flow through the soil via preferential paths, bypassing large parts of the soil matrix, e.g. [5, 6]. This reduces the availability of water and nutrients to plants, leaches chemicals such as pesticides [7] from the vadose zone to the groundwater, and causes accelerated transport of pollutants [8, 9].

Since preferential flow is a three-dimensional process, occurring at the scale of individual soil pores, it is difficult to map this process in the field. Using dye and/or tracer is, however, an efficient way to reveal spatial flow patterns though field soils. Using photographs of soil horizons and an appropriate algorithm to filter soil components covered and not covered with blue dye, the preferential flow of water can be visualized [10–12].

Many field studies using tracers have been conducted under high infiltration rates [13–18]. However, there is a lack of information for situations with lower infiltration rates. Several studies, e.g. [19, 20], have indicated constrained mobility of Brilliant Blue (BB) and showed that BB has limited capacity to serve as tracer for water flow in soils due to its sorption characteristics. However, BB behaves like many important organic contaminants (e.g., pesticides) and thus, provides information about concentration patterns at a much finer resolution as compared to other techniques [19]. On the other hand, bromide (Br^-^) is the most commonly used conservative tracer to monitor water movement in soil. As a negatively charged, non-reactive anion, it does not adsorb to negatively charged soil constituents, and it can be easily quantified in soil samples. Thus, by using both BB and Br^-^ in the same solution, a better representation of the water and solute transport can be captured. BB is believed to better mimic the movement of larger organic molecules, while Br^-^ is more appropriate for tracing water flow and nutrients such as NO_3_^-^N.

By combining dye with bromide, the retardation of dye can be quantified. Zehe & Flühler [21] combined BB and Br^-^ and found that the retardation factor ranges between 0.86 and 2.16. Öhrström et al. [10] found that in sandy soil (water content of about 0.30 m^3^ m^-3^) the retardation factor ranges from 1.47 to 1.5. Kasteel et al. [19] compared the mobility of BB in a field soil (Gleyic Luvisol) with that of bromide. They found that BB does not follow the same flow paths as bromide, but they did not repeat their experiments in different types of soil to test the difference in dye adsorption from soil to soil.

Although, tracer experiments are effective in capturing water and solute infiltration in unsaturated soil, they are time consuming and labour intensive. Further, they are destructive, the experiments can only be performed once at the same site. In addition, dye tracer experiments do not show flow dynamics. In combination with numerical simulation, these shortcomings can be overcome. Numerical simulation is a fast and efficient approach for simulating water and solute transport. Even though many researches have simulated soil water distribution under surface point source irrigation, e.g. [22–24], numerical simulation of the mobility of different tracers under drip irrigation has not been much studied. In view of the above, the aim of this study was to 1) investigate infiltration patterns with different tracers (bromide to mimic fertilizer and dye to mimic organic contaminant) under low infiltration rate in sandy soil, 2) study the potential preferential flow in dry sandy soil under point source irrigation, and 3) study the potential of using numerical model as a tool for rapid estimation of transport parameters (i.e., retardation factor) of different tracers under drip irrigation.

## Materials and methods

### Area description

The experiments were carried out in northern Tunisia at semiarid Nabeul located approximately 70 km southeast of Tunis. The experimental site was located at a research farm belonging to the Tunisian National Institute for Research in Rural Engineering, Water, and Forests (INRGREF). For the current experimental activities no specific permissions were required as the second author is a researcher at INRGREF. Moreover, the experimental field did not involve either endangered or protected species. The field site coordinates are 10^o^ 42' 16" E and 36^o^ 27' 44" N, and the altitude is 23.95 m amsl. The climate at the experimental site is Mediterranean, characterized by mild winters receiving the major part of the annual precipitation (450 mm on average) and hot and dry summers. Total rainfall and distribution are highly variable from year to year. Average annual potential evapotranspiration is 1370 mm [25].

The experimental plot was located at the first third of a 40 m x 40 m experimental field area. The groundwater table is found at about 4 m depth. Before the experiments, the field was tilled to a depth of 0.30–0.40 m. Drip irrigation is commonly used to irrigate vegetables and other crops in the area. At this particular site, drip irrigation was used one year before the experiments to irrigate potatoes. Three plots (N1, N2, and N3) were chosen with an inter-plot distance of 2.5 m and the dimensions of each plot were 2.0 m x 2.0 m.

### Field experiments

Local irrigation water with an electrical conductivity (σ_iw_) of 3.95 dS m^-1^ was used for the experiments. The irrigation water was mixed with BB dye (6 g *l*^-1^) and potassium bromide (4 g *l*^-1^), resulting in a total electrical conductivity (σ_p_) of about 10.5 dS m^-1^. The solute was applied through a single surface dripper with a constant average flux of 2.5 *l*h^-1^. This flux is typically used in the area when irrigating vegetables, e.g., tomatoes or cucumbers. Approximately 7.5 *l* was discharged from a small tank through the single dripper and a constant pressure was maintained using a small battery-driven pump.

The dye tracer used was the food-grade dye pigment Brilliant Blue (Swedish Hoechst Ltd.). This dye has been used in several studies due to its good visibility, low toxicity, and weak adsorption on soils [14, 26, 27, 11, 25, 28, 29]. The dye is readily soluble in water (solubility > 50 kg m^−3^) and the water solution gives a clearly visible blue staining to the soil and its electric conductivity is very low. After infiltration, the plots were covered with plastic sheet to avoid evaporation and to protect from rain. Fifteen hours after the infiltration, horizontal soil surface sections were dug with 5 cm intervals at each plot. A scale within a 50 cm by 50 cm wooden frame with its origin coinciding with the position of the dripper was put on the soil surface before taking photographs with a digital camera. The position of the frame was determined using two fixed points adjacent to each plot. Horizontal soil sections were photographed from 1.5 m height. A Sigma Probe (EC1 Sigma Probe, Delta-T Devices Ltd., Cambridge, UK) was used to measure soil solution electrical conductivity (σ_w_) at 5 cm horizontal intervals in a spatial grid within the 50 cm by 50 cm scale. The Sigma Probe measures σ_w_ independent of both soil moisture content (θ) and the degree of contact between the probe and soil [30–32]. The σ_w_ measurements were converted to relative electrical conductivity according to

$$\sigma_{rel}=\frac{\sigma_{w}-\sigma_{in}}{\sigma_{p}-\sigma_{in}}$$ (1)

where σ_in_ is the initial soil electrical conductivity and σ_p_ is the electrical conductivity of the applied infiltration pulse. Soil samples from different soil layers along the vertical axis below the dripper from surface to a depth of 60 cm in the three selected plots were collected to determine soil physical properties. Soil particle size was measured in the laboratory using the sedimentation method (pipette and hydrometer) and the gravimetric method was used to measure mass soil water content from the collected soil samples beneath the dripper to the depth of 60 cm before and after the infiltration experiment. A metallic ring was used to sample soil and measure the dry soil bulk density in order to estimate the volumetric soil water content. Table 1 shows the physical properties of the different soil layers at the three experimental plots. The soil texture is classified as loamy sand.

**Table 1:** Soil bulk density and particle size distribution of the experimental plots.

Plots	Bulk density (g/cm^3^)	Fine silt %	Coarse silt %	Fine sand %	Coarse sand %
N 1					
0–10	1.65	7.0	3.0	36.5	53.5
10–20	1.64	8.5	3.5	41.0	47.0
20–40	1.64	10.5	2.5	21.0	66.0
40–60	1.66	14.0	3.0	31.0	52.0
N 2					
0–10	1.62	9.0	7.0	40.0	44.0
10–20	1.68	13.0	4.5	42.5	40.0
20–40	1.71	9.0	5.0	38.0	48.0
40–60	1.72	7.0	5.5	38.5	49.0
N 3					
0–10	1.52	12.0	3.0	27.5	57.5
10–20	1.47	11.0	5.5	45.0	38.5
20–40	1.74	10.0	5.0	43.0	42.0
40–60	1.81	10.0	2.5	17.5	70.0

### Image analysis

The digitized images were analyzed using Adobe Photoshop (Adobe Systems Inc.). The images were converted into the CMYK (Cyan, Magenta, Yellow, and Black) colour space. The cyan channel was chosen for recognizing stained from unstained soil and the remaining channels were discarded. After that, the images were transferred into gray-scale. A histogram for each image was made to achieve a gray level threshold that discriminated coloured soil from non-coloured soil. By using the image processing toolbox in Matlab (The Mathworks Inc.), the images were converted into black and white images with a resolution of 0.3 cm x 0.3 cm and the dye covered area was calculated. For more details, see Selim et al. [25]. The dye-covered area was calculated in order to estimate the bromide and dye volumetric retardation factors. In general, soil sections were excavated until no dye traces could be observed. This meant in most cases down to a depth of 50 cm and an average of eleven images at each plot.

### Water repellency experiments

Sand grains in sandy soils (sands to sandy clay loams) sometimes become water repellent by the coating of organic residues from plant materials. The rate of disappearance of drops of water on a soil surface is used as a method of determining the severity of water repellency [33]. Water repellency experiments were done by two methods. In the first method, a core sampler, 1.5 cm in diameter, was used to collect undisturbed soil samples to a depth of 50 cm. Water drop penetration test was conducted at every 2 cm soil depth. In the second method, experiments were conducted at the plot surface at a depth of 5 cm from soil surface. The procedure was as follows (see e.g. [34, 35]):

1. A droplet of deionised water (approximately 6 mm diameter) was dropped from a height of 1.5 cm on the surface of the soil.
2. If the droplet adopts a spherical shape on the soil surface, water repellency is indicated.
3. The water drop penetration time (length of time at which the droplet remains on the surface) was recorded. This time is taken as an index and quantification of water repellency (Table 2).

Each experiment was repeated twice for each soil depth at two points separated by 3 cm.

**Table 2:** Classification of water repellence according to water drop penetration time.

Time	Water repellency
< 1 second	Not significant
1–10 seconds	Very low water repellency
10–50 seconds	Low water repellency
50–260 seconds	Moderate water repellency
> 260 seconds	Moderate to severe water repellency

### Numerical simulation

The solute mobility of the dye and bromide under surface drip irrigation was simulated with two-dimensional numerical modelling using HYDRUS-2D/3D software package [36]. The HYDRUS software package simulates two and three-dimensional movement of water, heat, and multiple solutes in variably saturated media based on finite-element numerical solution of the flow, mass transfer, and heat equations. Assuming a homogeneous and isotropic soil, the program uses the Galerkin finite-element method to solve the 2D Richards’ equation [37] for saturated-unsaturated water flow and solute transport with the convection-dispersion equation, e.g. [38]. For more details about HYDRUS code and its applications, see [36].

**Fig 1:**
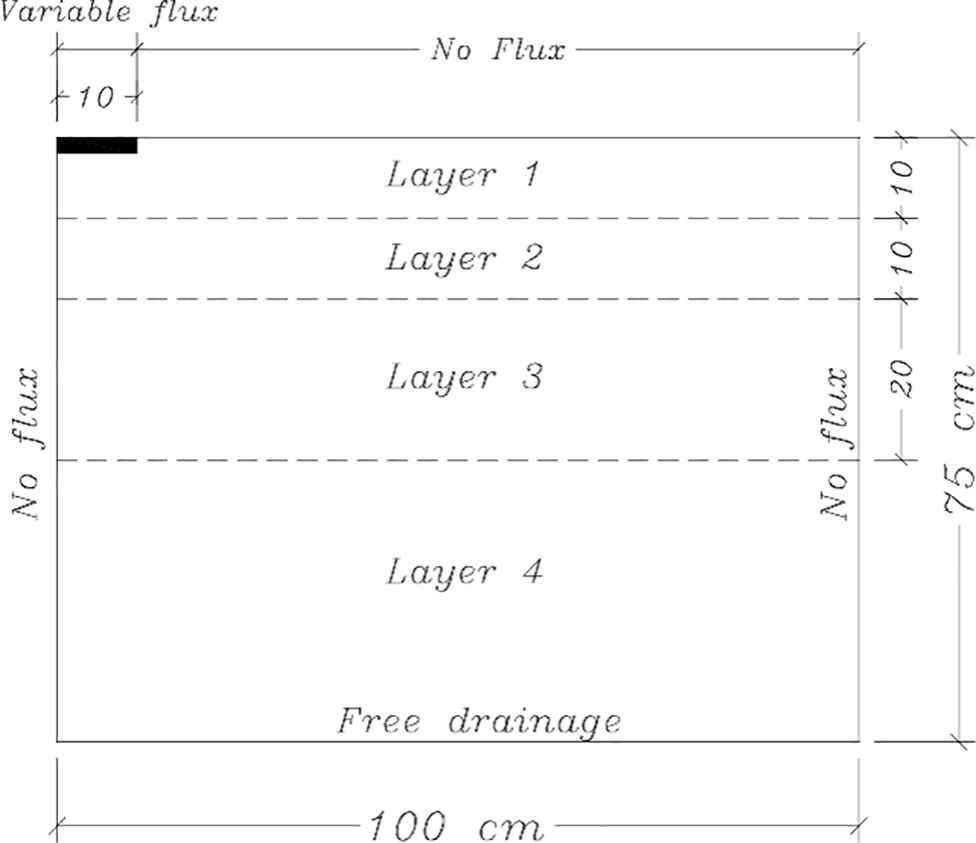
Conceptual diagram of simulated area.

The simulated domain was axi-symmetrical, 100 cm width and 75 cm depth (one-half of the flow domain). Unstructured triangular mesh with 5617 2D elements was used to spatially discretize the flow domain. Triangular elements of smaller sizes were generated closer to the soil surface where rapid change in flux occurs. The simulation assumed no flux boundary conditions along the vertical sides of the soil domain. Bottom boundary was considered as free drainage boundary because the water table is situated far below the domain of interest (4 m below the soil surface). Because of covering the plots with plastic sheet during the field experiment, a no flux top boundary was assumed throughout the simulation period except during the period of water application. Water application was constant flux of 7.95 cm hr^-1^ at the location of dripper. The flux area was the area of a circle with radius of 10 cm. This radius was taken as neither ponding nor surface runoff was assumed to occur. Fig 1 shows the conceptualized simulated area and the imposed boundary conditions.

Longitudinal dispersivity (ε_L_) was approximated to one-tenth of the profile depth (see e.g. [39, 40]). The transversal dispersivity (ε_T_) was set equal to 0.1 ε_L_. The values of ε_L_ rely on measurements scale. The influence of measurement scale on ε_L_ was reported by Phogat et al. [41]. They stated that ε_L_ varies from 0.5 to 2 cm for laboratory experiments with packed columns while in intact columns and field soils it ranges from 5 to 20 cm. On the other hand, it exceeds 1 m in groundwater flow through aquifers. The dye adsorption isotherm coefficient was set equal to 0.10 cm^3^ g^-1^ [10], and molecular diffusion coefficients in free water were 0.0738 and 0.0036 cm^2^ h^-1^ for bromide and dye, respectively. The initial θ distribution within the flow domain was chosen related to the field measurements. The simulations were conducted for an 18-hr infiltration period.

### Soil hydraulic parameters optimization by inverse modelling

Appropriate estimation of soil hydraulic parameters provides good simulation for the field data. Based on the measured water content within the soil profile before and after the infiltration experiment, inverse parameter estimation option (using Marquardt-Levenberg parameter optimization algorithm) in HYDRUS-2D/3D was used to identify soil hydraulic parameters. Initially, soil hydraulic parameters (residual water content, *θ*_*r*_; saturated water content, *θ*_*s*_; saturated hydraulic conductivity, K_s_; shape parameters, α and n; and pore connectivity parameter, *l*) for different soil layers were assumed using Rosetta pedotransfer function of Schaap et al. [42], using the observed soil particle size percentage and soil bulk density, to execute the inverse solution. After that, predicted and observed soil water contents were compared and the final soil hydraulic parameters were selected to minimize this difference.

## Results and discussion

### Water repellency

Table 3 shows results of the water repellency experiments with soil depth and Table 4 in the horizontal direction with one axis passing through the plot at the soil surface. The results clearly show that the water drop penetration time is very small and that water repellency is very low. Thus, water repellency appears not to be a reason for heterogeneous solute transport at the investigated field sites.

**Table 3:** Water drop penetration time with soil depth.

Soil depth (cm)	T_1_ (s)	T_2_ (s)	Soil depth (cm)	T_1_ (s)	T_2_ (s)
0	1.70	1.60	26	0.90	0.90
2	1.20	1.40	28	1.10	1.20
4	1.10	1.20	30	1.12	1.14
6	1.20	1.30	32	0.80	1.00
8	1.50	1.12	34	0.80	1.10
10	0.80	0.50	36	1.40	1.60
12	0.60	0.40	38	1.10	1.30
14	1.08	1.05	40	1.40	1.50
16	1.80	1.70	42	1.40	1.60
18	1.00	0.80	44	1.40	1.80
20	0.80	0.90	46	1.20	1.30
22	1.10	1.20	48	1.50	1.40
24	1.35	0.80			

**Table 4:** Water drop penetration time in horizontal direction of soil surface.

Distance (cm) at one axis	T_1_ (s)	T_2_ (s)
0	1.40	1.40
5	1.70	1.70
10	1.10	1.50
15	1.10	1.50
20	1.30	1.90
25	2.40	2.40
30	2.90	2.70
35	1.20	1.70
40	2.60	1.90
45	1.90	1.70
50	2.20	2.10

### Dye and bromide analysis

**Fig 2:**
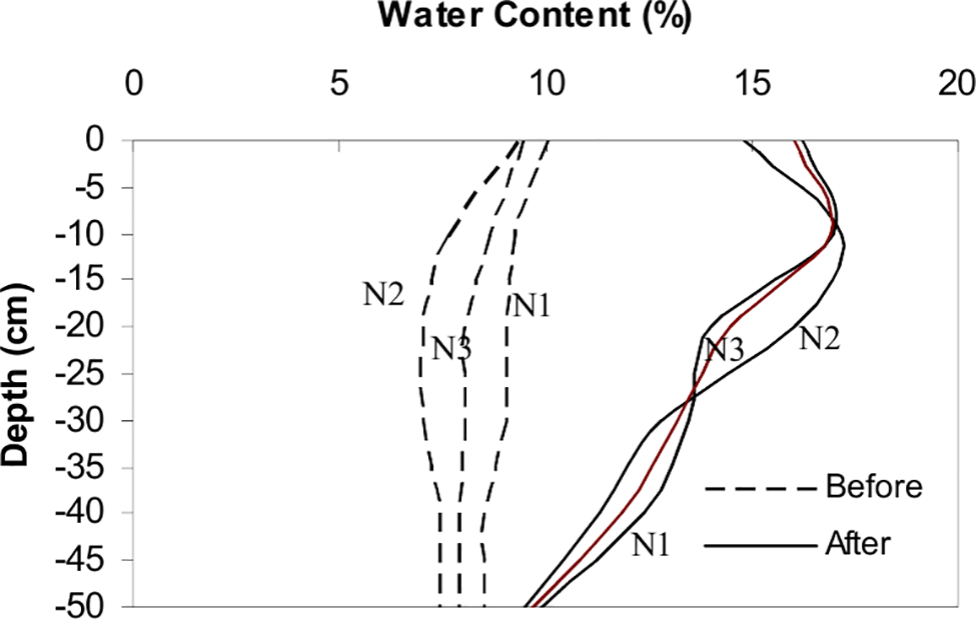
Soil water content before and after infiltration below the dripper.

Fig 2 shows soil water content directly beneath the dripper before and after infiltration for the three plots. The initial soil moisture content varied from 0.074 to 0.10 m^3^ m^-3^. The average content of clay, silt, and sand was 0, 14, and 86% respectively (Table 1). Fifteen hours after ceasing the water application, θ was higher at the top 10 cm of the soil profile. This indicates that the redistribution process continued. Due to the low initial θ value, the soil colour was relatively light and the dyed areas were easily distinguished.

**Fig 3:**
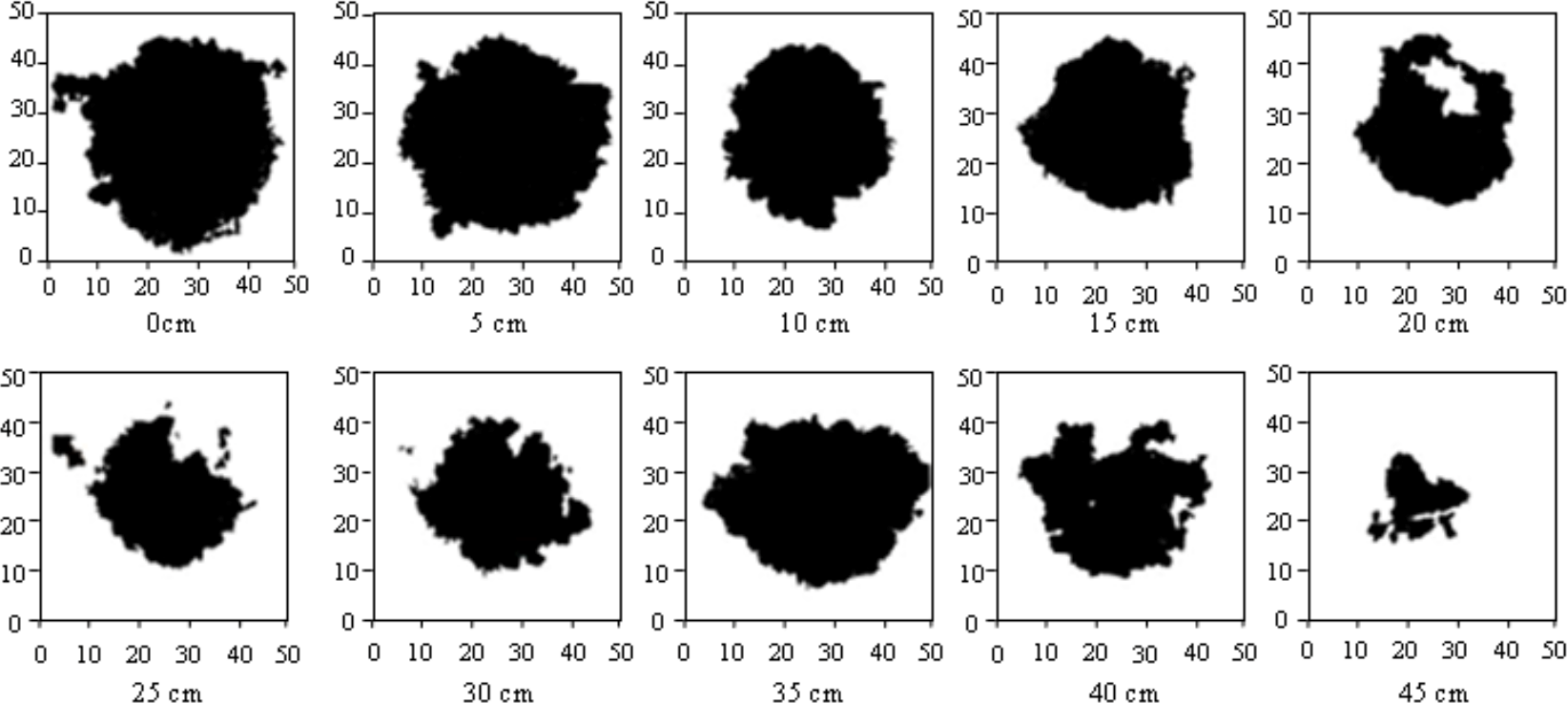
Dye patterns with depth in sandy soil (plot N2), no dye was observed at the 50 cm depth. Horizontal sections are 50 cm in length and 50 cm in width with dripper in the centre.

The dye patterns with depth at each of the three plots had approximately the same shape. The maximum dye penetration was 45 cm in plots N1 and N2. In plot N3 the maximum dye penetration was 50 cm. The greater dye penetration depth at plot N3 was probably caused by a higher percentage of coarse sand at deeper layer (70% at 40–60 cm, see Table 1). The dye pattern with depth for plot N2 is shown in Fig 3. This dye pattern is representative for all three plots. As mentioned above, water drop penetration time was very low for all plots. Thus, observed heterogeneity is likely not caused by water repellency. Observed heterogeneity for the solute transport is probably caused by other factors such as occurring ped structure and small-scale differences in hydraulic conductivity. In general, however, observed solute transport heterogeneity was dominated by small-scale effects and there was no evidence of deep preferential flow. Consequently, for the investigated soil, drip irrigation does not appear to induce large solute transport heterogeneity.

**Fig 4:**
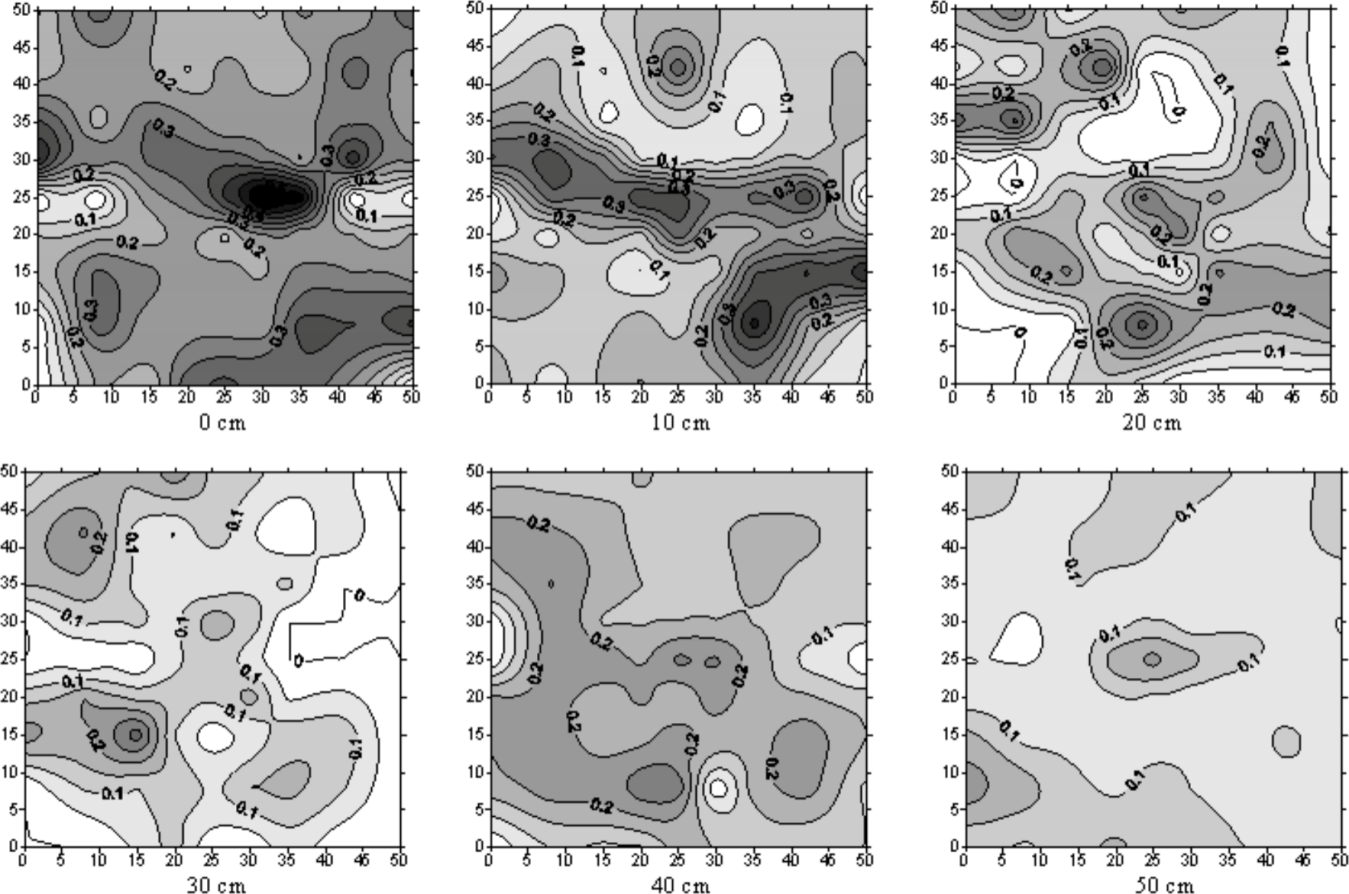
The isolines of relative bromide concentration in different horizontal sections (plot N2). Horizontal sections are 50 cm in length and 50 cm in width with dripper in the centre.

Fig 4 shows the isolines of relative bromide concentration at different horizontal sections (10 cm interval) for plot N2. Bromide was detected in an area larger than the 50 cm by 50 cm frame. Unfortunately, no measurements were taken outside the frame. However, visual inspection revealed that the wetted area only extended slightly outside the frame for most depths. Thus Fig 4 gives an almost complete picture of the bromide distribution. The isolines of bromide represent detected bromide concentrations by the sigma probe. The isolines in the measured profiles were drawn using a kriging interpolation algorithm. Outside the outer isoline, the bromide concentration was too low or the soil was too dry to allow for measurements. The actual initial σ_w_ could not be observed because the sigma probe does not function in dry soil (θ < 0.10 m^3^m^-3^). When plotting the isolines, the relative bromide concentration was manually set equal to zero when the sigma probe gave the "too dry" error message.

According to the relative bromide concentration, isolines at the 20 cm depth in plot N2, it can be noted that the σ_rel_ = 0 coincides with the unstained patch in the dye pattern at the same depth. Consequently, at this point bromide concentration and dye pattern agree well. In almost all horizontal sections for all plots, the maximum relative bromide concentration was detected just beneath the dripper. Some higher relative bromide concentrations were detected far from the dripper that may be attributed to occasional preferential flow in the top tillage soil layer. From the relative bromide concentration isolines at all plots, it was observed that the concentration of bromide is less than the concentration of the applied pulse. This shows the solute dispersion as the solute pulse migrates through the soil. It should be noted that the soil was initially dry, which means that no sigma probe readings could be taken. Consequently, observed conductivity readings in the unstained soil indicate that the soil was wetted (water and bromide reached this soil part but not the dye). And thus, this is evidence that the bromide flow is different as compared to the dye. Comparing the patterns of both relative bromide concentration and dye distribution, it was observed that the relative bromide concentration distribution had larger heterogeneity than dye. This can be explained by different dispersion properties and solute sorption between dye and bromide. However, since the dye was only recorded as stained or unstained soil, areas with higher dye concentration could not be identified.

**Fig 5:**
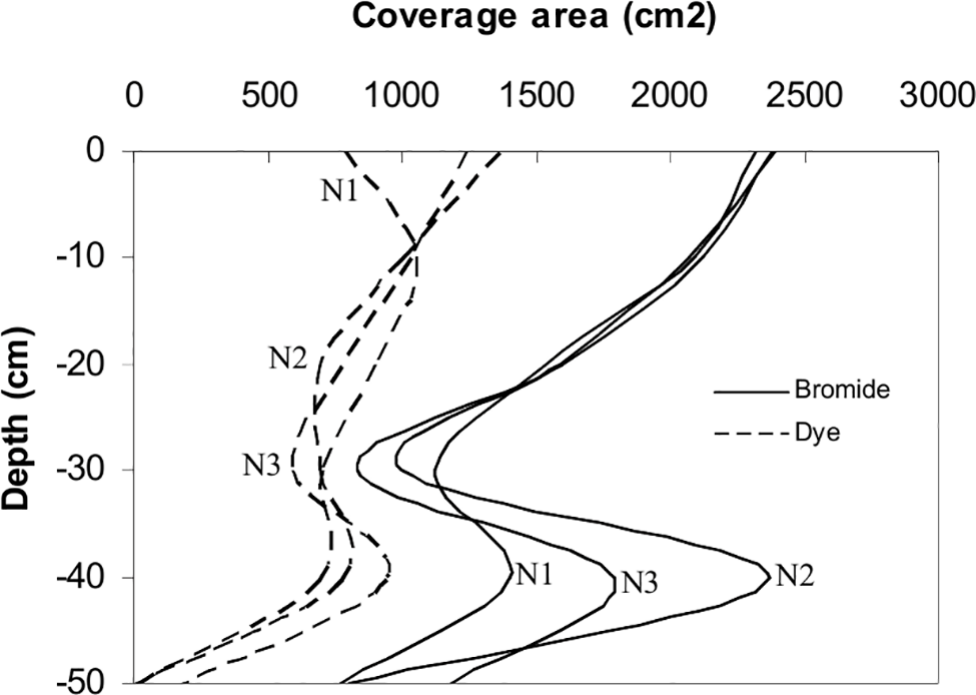
Coverage area for both dye and bromide with depth.

Fig 5 shows the dye-bromide covered soil area with depth. From the dye coverage area curve, it can be noted that there are two peaks, one upper at the soil surface layer and one deeper at about 30–40 cm depth. The first upper peak is probably due to the near-saturated conditions close to the dripper and thus possibilities for horizontal transport in soil close to the soil surface. The second deeper peak demarks the tillage depth. The transition between the upper tilled and lower untilled soil layers caused horizontal flow. Comparing the impact of textural soil stratification on water flow, Hillel [43] concluded that it is soil layer with lower hydraulic conductivity that controls the process.

The area covered by bromide at different horizontal sections was estimated from the Sigma Probe readings corresponding to relative bromide concentration higher than 0.10. Öhrström et al. [10] found that the visible lower limit of dye in a loamy sand soil corresponds to a relative bromide concentration of 0.10 (using similar dye pulse concentration). Consequently, it is clearly seen that the dye was retarded in relation to bromide in both vertical and horizontal directions.

### Retardation factors

The above results were used to estimate dye-bromide volumetric retardation factors. In general, BB has similar adsorptive behaviour as, e.g., organic contaminants while bromide ions move more like conservative substances, e.g., fertilizers [19, 44]. Thus, by using BB and bromide, a rough but general idea on how fertilizers and other contaminants may be transported in the present initially low water content soil type can be established. To quantify the difference in retardation, the volumetric retardation factor (R_vol_) between bromide and dye was calculated. This was achieved by dividing the volume of soil with measurable bromide concentration by the volume of soil stained with dye

$$R_{Vol}=\frac{\mathrm{Volume}\;\mathrm{of}\;\mathrm{soil}\;\mathrm{stained}\;\mathrm{by}\;\mathrm{bromide}}{\mathrm{Volume}\;\mathrm{of}\;\mathrm{soil}\;\mathrm{stained}\;\mathrm{by}\;\mathrm{dye}}$$ (2)

The volume of soil stained by both bromide and dye was calculated by integrating the area under bromide-dye coverage area curve (Fig 5). The retardation factor R is related to the adsorption k_d_ by

$$R=1+\frac{\rho_{b}}{\theta }k_{d}$$ (3)

Different methods have been envisaged for calculating the adsorption coefficient (e.g., [45–47]). In our study, R_vol_ was found to be 1.98, 2.04, and 1.95 for plot N1, N2, and N3, respectively. These results concur with results in previous studies for soils with similar texture. A retardation factor of 2.00 corresponds to k_d_ of 0.10 dm^3^ kg^-1^ (for ρ = 1.68 gm cm^-3^, *θ* = 0.17 m^3^ m^-3^).

### Numerical simulation analysis

#### Soil parameter optimization

Table 5 shows the soil hydraulic parameters for different soil layers used to simulate solute transport through the soil. These parameters are result of running the inverse parameter estimation option in HYDRUS-2D/3D depending on the soil water content measurements directly below the dripper by the end of infiltration experiment.

**Table 5:** Hydraulic parameters of simulated soil layers for plot N2.

Soil layer	Depth (cm)	Ө_r_ (m^3^ m^-3^)	Ө_s_ (m^3^ m^-3^)	α	n	k_s_ (cm/h)
1	0–10	0.010	0.325	0.049	1.365	32.8
2	10–20	0.026	0.491	0.054	1.516	18.8
3	20–40	0.013	0.508	0.052	1.640	35.3
4	40–75	0.028	0.375	0.059	1.660	21.3

**Fig 6:**
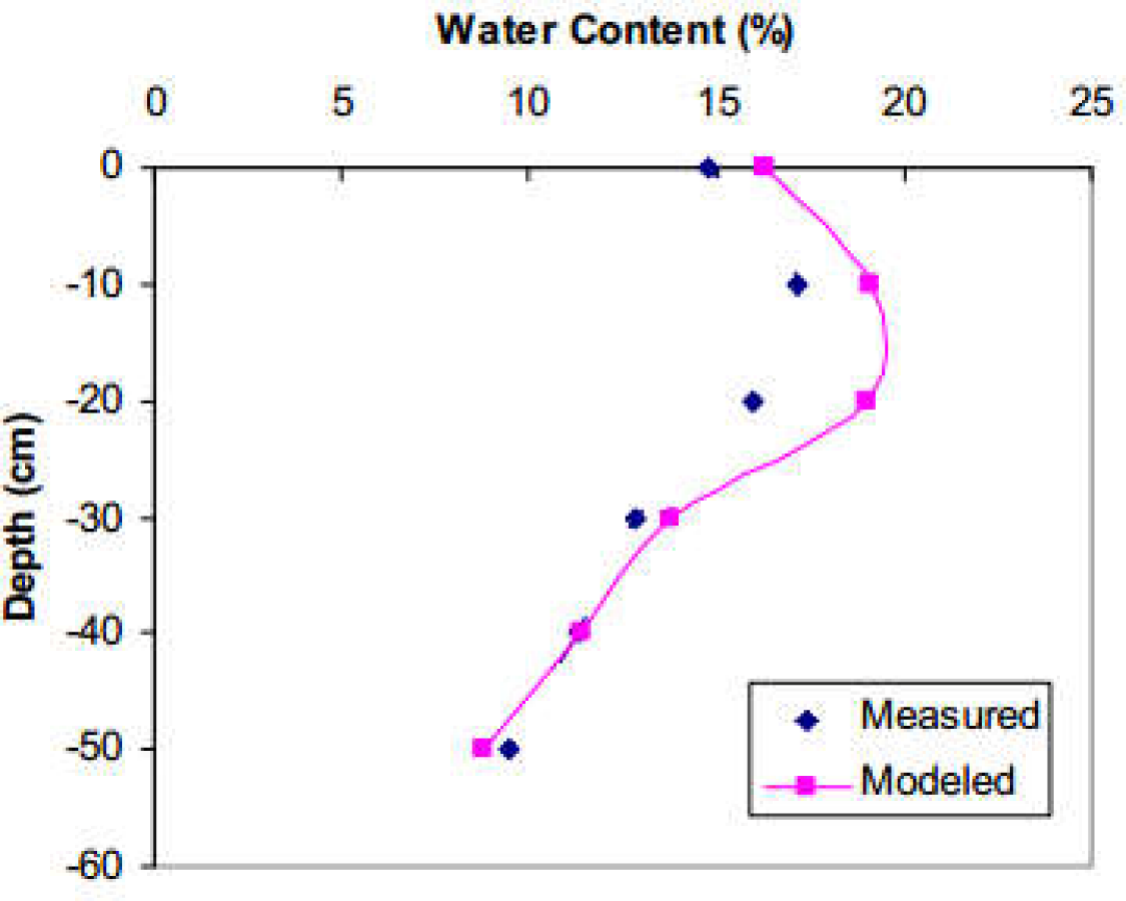
Comparison between measured and simulated water content profile under the dripper (plot N2).

As seen from Fig 6, the predicted water content distribution agrees in general rather well with observations. The model, however, overestimates water content in the upper tilled layer. This is caused by a faster redistribution process in the tilled top soil layer in the field, while the soil in the model was assumed to be homogenous. The root mean square error (RMSE) corresponds to 0.016 m^3^ m^-3^ and the coefficient of determination (R^2^) to 0.91 between the simulated and observed volumetric soil water content. In general, this indicates acceptable simulation results.

#### Bromide and dye transport

**Fig 7:**
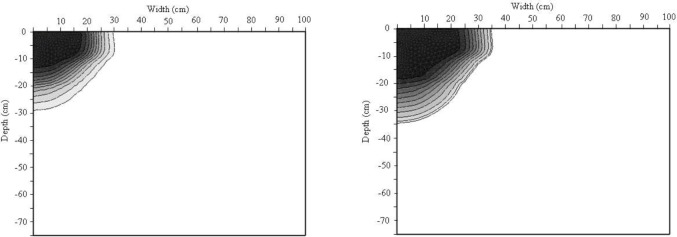
Contour maps for dye and bromide concentration of plot N2 (dye in the left and bromide in the right).

HYDRUS-2D/3D was used to investigate the transport of bromide and dye through the soil. Fig 7 shows contours of dye concentration larger than 0.2 g *l*^-1^. Dye at concentration lower than 0.2 g *l*^-1^ could not be analyzed by the image processing tool, e.g. [48, 10]. Fig 7 also shows contours for σ_rel_ larger than 0.10. From these graphs, it is clear that the mobility of dye differs substantially from that of bromide.

The difference in simulation results is mainly due to the different adsorption characteristics. The bromide-dye volumetric retardation factor was calculated using Eq (2). The volume of soil stained by bromide was calculated based on the hypothesis that the bromide stains a soil volume equal to a half sphere with radius equal to the bromide infiltration depth beneath the dripper. The same calculations were carried out for the dye. The volumetric retardation factor was thus, found to be 1.93, 1.85, and 1.80 for plots N1, N2, and N3, respectively. These results are close to the retardation factors for the field measurements described above. It is worth noting that the difference between measured and simulated depths for both bromide and dye may be due to the difference in nature between field and simulated soil regarding soil heterogeneity. Although, vertical heterogeneity was considered in simulation by dividing the simulation domain into four layers, each individual layer was assumed homogenous in simulation that obviously was not the case in the field soil. In addition, the assumed dispersivity in the numerical model may be a source of error in simulations. Although, bromide has ability to describe water movement in the vadose zone, the simulated bromide penetration depth is less than the maximum simulated wetted depth. This is probably due to the diffusion and dispersion characteristics of bromide that lead to very low concentrations at the wetting front.

## Summary and conclusions

Field experiments in a sandy soil were done to investigate solute transport patterns from a single dripper. At each plot about 7.5 *l* dye and bromide tagged irrigation water was infiltrated at approximate constant rate of 2.5 *l* hr^-1^. This is a typical daily application rate in the area using drip irrigation. Preferential flow occurred to some degree, however, did not dominate the solute transport process. The coarse soil particle size distribution and the near absence of soil aggregates explain these results. Consequently, the lack of preferential flow is an advantage for better water irrigation efficiency and soil leaching. Because of the coarse sandy soil, drip irrigation can be recommended to improve plant culture for a better water and soil nutrient adsorption.

The observed differences in retardation between bromide and dye can be used as an initial value for retardation of fertilizers and organic pollutants, respectively. Numerical simulation with good estimation of soil hydraulic properties for the field experiment showed consistent results for the retardation factor.

In sum, the simulation results for solute mobility were in a good agreement with field observations. Therefore, HYDRUS-2D/3D can be used as a tool for rapid estimation of transport parameters (retardation factor) in sandy soil under point source irrigation. Furthermore, it facilitates the identification of contaminant transport on the surrounding environment.
